# Variability in Host Specificity and Functional Potential of Antarctic Sponge-Associated Bacterial Communities

**DOI:** 10.3389/fmicb.2021.771589

**Published:** 2022-01-13

**Authors:** Antonia Cristi, Génesis Parada-Pozo, Felipe Morales-Vicencio, César A. Cárdenas, Nicole Trefault

**Affiliations:** ^1^Centro GEMA – Genómica, Ecología y Medio Ambiente, Facultad de Ciencias, Universidad Mayor, Santiago, Chile; ^2^Department of Marine Science, University of Otago, Dunedin, New Zealand; ^3^National Institute of Water and Atmospheric Research, Wellington, New Zealand; ^4^Departamento Científico, Instituto Antártico Chileno, Punta Arenas, Chile; ^5^Millennium Institute Biodiversity of Antarctic and Subantarctic Ecosystems (BASE), Santiago, Chile

**Keywords:** Antarctic sponges, symbiosis, high-throughput sequencing, 16S rRNA gene, microbiome, host specificity, functional potential, secondary metabolites

## Abstract

Sponge-associated microorganisms are essential for sponge survival. They play an important role in recycling nutrients and, therefore, in the maintenance of the ecosystem. These microorganisms are diverse, species-specific, and different from those in the surrounding seawater. Bacterial sponge symbionts have been extensively studied in the tropics; however, little is known about these microorganisms in sponges from high-latitude environments. Sponges can cover up to 80% of the benthos in Antarctica and are crucial architects for the marine food web. In this study, we present analyses of the bacterial symbionts of three sponges: *Haliclona (Rhizoniera)* sp., *Hymeniacidon torquata*, and *Isodictya kerguelenensis* from the Western Antarctic Peninsula (WAP) with the aim to determine variations on the specificity of the bacteria–sponge interactions and potential signatures on their predicted functional profiles. We use high-throughput 16S rRNA gene sequencing of 30 sponge individuals inhabiting South Bay (Palmer Archipelago, WAP) to describe their microbiome taxonomy and diversity and predict potential functional profiles based on this marker gene. Our work shows similar bacterial community composition profiles among the same sponge species, although the symbiotic relationship is not equally conserved among the three Antarctic sponges. The number of species-specific core operational taxonomic units (OTUs) of these Antarctic sponges was low, with important differences between the total abundance accounted for these OTUs. Only eight OTUs were shared between the three sponge species. Analyses of the functional potential revealed that despite the high host–symbiont specificity, the inferred functions are conserved among these microbiomes, although with differences in the abundance of specific functions. *H. torquata* showed the highest level of intra-specificity and a higher potential of pathways related to energy metabolism, metabolisms of terpenoids and polyketides, and biosynthesis of other secondary metabolites. Overall, this work shows variations in the specificity of the sponge-associated bacterial communities, differences in how hosts and symbionts establish their relations, and in their potential functional capabilities.

## Introduction

Sponges are sessile and filter-feeding metazoan, which host a great number and diversity of microorganisms from the three domains of life in a symbiotic relation. These microorganisms enhance the host fitness and survival; they contribute to the sponge energy requirements through nitrogen fixation and photosynthesis and defend against predators and epibionts by synthesizing secondary metabolites (Webster and Taylor, 2012; Webster and Thomas, 2016). Sponges and their associated microorganisms are considered holobionts, which give the sponge the characteristic of an ecosystem capable of resilience to environmental perturbations (Pita et al., 2018). Sponge holobionts are fundamental for the maintenance of the food web, and they circulate dissolved organic matter through the sponge loop, participate in the nitrogen and phosphorus biogeochemical cycling, and fulfill important roles for the benthopelagic coupling due to their high filtering capacity (Bell, 2008; Fiore et al., 2010, 2015; De Goeij et al., 2013; Zhang et al., 2015).

Sponges are classified as “high microbial abundance” (HMA), which can host up to 10^9^ cells/cm^3^, or “low microbial abundance” (LMA), hosting 10^5^ to 10^6^ cells/cm^3^, based on the number of bacterial cells associated with them (Webster and Thomas, 2016). Despite this binary classification, the symbiotic relation is not equally complex across sponge species and is mainly shaped by environmental factors and the host phylogeny (Thomas et al., 2016). Moreover, multiple ecological and evolutionary processes acting both within host and among microbes could also modulate host–microbiome interactions (Adair and Douglas, 2017). The relationship between sponges and their bacterial symbionts is species specific and acquired *via* vertical transmission (Hentschel et al., 2002; Schmitt et al., 2008). However, sponges contain a common set of functional genes even in distantly related sponges (Burke et al., 2011; Ribes et al., 2012). The high degree of host specificity (Taylor et al., 2004; Thiel et al., 2007; Hardoim and Costa, 2014; Reveillaud et al., 2014) relies on molecular signatures that allow symbiotic lifestyle (Gao et al., 2014; Zhang et al., 2019). However, recent studies have shown that HMA sponges host more similar bacterial symbionts and a higher degree of diversity and evenness in their composition than LMA sponges (Erwin et al., 2015; Turon et al., 2018).

Furthermore, it has been observed that the processes behind vertical transmission in sponges are both neutral and selective (Björk et al., 2019). This means that a set of symbionts is transmitted from parents to larvae and that there is also a selection over this transmission. Altogether, these studies demonstrate that the acquisition process of bacterial symbionts by sponges is more complex than initially thought and that the establishment of the symbiotic relationship depends on multiple factors.

Despite the great importance of sponges in the Antarctic benthos, with ∼390 species described (Downey et al., 2012), and the pivotal role sponge microorganisms play for sponges, there are only a few studies about microbial symbionts in Antarctic sponges using high-throughput molecular approaches (Rodríguez-Marconi et al., 2015; Cárdenas et al., 2018, 2019; Savoca et al., 2019; Steinert et al., 2019; Sacristán-Soriano et al., 2020; Moreno-Pino et al., 2021). These studies revealed some particularities of sponge symbionts in this environment, such as the absence of Cyanobacteria and Poribacteria, bacterial phyla typically identified in tropical sponges, and the dominance of Proteobacteria (mainly alpha and gamma) and Bacteroidetes (Rodríguez-Marconi et al., 2015; Cárdenas et al., 2018, 2019). In addition, it also confirmed the high degree of host specificity in this environment (Sacristán-Soriano et al., 2020). Studies using culture-dependent approaches in sponges from the Ross Sea and the Western Antarctic Peninsula (WAP) confirmed the presence of Gammaproteobacteria as the dominant bacterial class while also supporting the hypothesis of microbial selection through filtering in these sponges (Savoca et al., 2019; Moreno-Pino et al., 2021). Furthermore, carbon, nitrogen, sulfur, and phosphorus cycling was detected in two abundant sponges from the WAP—*Leucetta antarctica* and *Myxilla* sp.—including the presence of pathways for light-independent carbon fixation mediated by chemoautotrophic microorganisms (Moreno-Pino et al., 2020). These findings suggest that the sponge microbiome plays an essential role in the host survival in this environment.

In this study, we present the analysis of the bacterial symbionts of three sponge species (*Haliclona (Rhizoniera)* sp., *Hymeniacidon torquata*, and *Isodictya kerguelenensis*) from the WAP to understand their intra- and inter-species microbiome variability. To determine whether variability in community composition within and between the sponge species is maintained at the functional level, we infer their functional potential profiles, focusing on energy metabolism and biosynthesis of secondary metabolites. We included several replicates of each sponge species to prove that the relationship between the sponge microbiome and host species is not equally conserved among different Antarctic sponges.

## Methodology

### Sample Collection

Sponge individuals of three Demospongiae species were collected from Cape Kemp at Doumer Island, Western Antarctic Peninsula (64°51′58.6″S, 63°37′46.7″W) during the Austral summer of 2016. Ten individuals of *Haliclona (Rhizoniera)* sp. (order Haplosclerida, family Chalinidae), 15 individuals of *H. torquata* (order Suberitida, family Halichondriida), and five individuals of *I. kerguelenensis* (order Poecilosclerida, family Isodictyidae) were selected for the analyses. All three are common sponge species in this study area (Fernandez et al., 2020). Sponge samples were collected by SCUBA divers between 10 and 20 m depth. Seawater temperature ranged from 0.6 to 1.1°C. See Supplementary Table 1 for detailed sponge sample information, each sponge individual used in this study was identified with an R (for replicate) followed by a number. Sponge samples were kept individually in plastic bags containing natural seawater at 4°C until processing within a few hours after the collection.

### Sponge Treatment and DNA Extraction

Each sponge individual was rinsed three times with sterilized seawater, carefully cleaned under a stereomicroscope to remove dirt and ectoparasites, and stored with RNA later at 4°C for 24 h and then at −20°C until processing. Triplicate tissue samples of ∼0.5 cm^2^ were extracted with a sterile scalpel blade from each sponge sample. The microbial community associated with the sponge was separated according to the protocol of Rodríguez-Marconi et al. (2015). DNA from the microbial community was extracted using the PowerSoil DNA Isolation Kit (MOBIO).

### High-Throughput Ribosomal Gene Sequencing

We amplify the hypervariable region V4 of 16S rRNA for high-throughput ribosomal gene sequencing, using F515 (GTGYCAGCMGCCGCGGTAA) and R806 (GGACTACHVGGGTWTCTAAT) primer pair (Caporaso et al., 2011). Three independent PCR amplicons were generated for each sponge individual. PCR reactions were performed in 35-μl final volume with Taq buffer 1 × final concentration, 2 nM of MgCl_2_, 0.3 nM of dNTPs, 0.3 μM of each primer, 2.5 units of GoTaq Flexi DNA Polymerase (Promega), and 1–6 ng of template DNA. Amplification conditions were 10 min of initial denaturation at 94°C, 28 cycles of 94°C for 30 s, 55°C for 1 min, and 72°C for 1.5 min, followed by a final extension of 72°C for 10 min. Illumina primer constructs were obtained from the Earth Microbiome Project (Gilbert et al., 2014). The three amplicons generated from the same sponge sample were combined and quantified using a standard qPCR assay using a Library Quant Kit Illumina (Kapa) according to manufacturer instructions. Finally, these combined amplicons were equimolarly pooled and sequenced using Illumina MiSeq following the protocol of Caporaso et al. (2011) and a 300-cycle Illumina MiSeq kit.

### Sequencing Data Analysis

Data analysis was performed using Mothur software v.1.38.1 (Schloss et al., 2009). Initial reads were assembled using the function *make.contigs*. Reads were cleaned by removing primer sequences using Cutadapt software. Sequences less than 252 bp and with homopolymers longer than 8 and maximum ambiguous longer than 0 were removed using *screen.seqs*. Singleton reads were removed using *split.abund* with a cutoff of 1. The alignment was performed using a customized database from the recreated SILVA SEED database v132 for archaea and bacteria using a threshold of 0.8. Chimera detection and removal were carried out using *chimera.uchime*. Sequences with <10 reads were discarded from further analysis. Operational taxonomic units (OTUs) were built at 97% similarity with the furthest neighbor algorithm implemented by Mothur and assigned using Greengenes database gg_13_5_99. Sequences were filtered using *remove.lineage* for chloroplast, mitochondria, and unknown and unclassified taxa at the class level. Taxonomic assignments of OTUs representing 70% of relative abundance were confirmed using the NCBI database version 231 (March 05, 2019).

Microbiome analysis was conducted using Phyloseq (McMurdie and Holmes, 2013). Reads by sample were normalized using median sequencing depth. Taxonomy plots and unconstrained principal component analysis (PCA) were done at the order level using MicroViz (Barnett et al., 2021), and taxa were center log-transformed using the function *clr*. Shannon diversity index was determined using Mothur. Sponge core OTUs were determined based on a 100% threshold, meaning that only OTUs present in all individuals from the same sponge species were considered core. “Species-specific core OTUs” were defined as OTUs present in all individuals of the same sponge species, while “shared core OTUs” are core OTUs present in all individuals of all sponge species. Upset plots were done in R using the UpSetR package. To determine differences among community composition between sponge species, a non-parametric PERMANOVA (NPMANOVA) was performed using PAST version 2.17c with OTUs that represent 90% of the abundance as variable and 9,999 permutations. In addition, one-way ANOVA and Tukey’s honestly significant difference (HSD) were done in R using the function *aov* and *TukeyHSD*.

### Prediction of Sponge Microbiome Functional Potential

The functional potential in the sponge microbiomes was predicted using Phylogenetic Investigation of Communities by Reconstruction of Unobserved States (PICRUSt) according to Hutlab Galaxy version 1.0.0 (Langille et al., 2013). PICRUSt analysis determines predicted KEGG functional categories using the 16S rRNA gene abundances among microbial communities and representative public genome information. In addition, the nearest sequenced taxon index (NSTI), a value that expresses the availability of representative genomes for each sample, was determined as quality control.

Functions were predicted until KEGG pathway hierarchy level 3, corresponding to 326 KEGG orthologs distributed in five categories: cellular processes, environmental information processing, genetic information processing, organismal systems, and metabolism. We focus on the metabolism category, which has 12 sub-categories. We look deeper into energy metabolism, metabolisms of terpenoids and polyketides, and biosynthesis of other secondary metabolites.

## Results

### Diversity Patterns in Antarctic Sponge Microbiomes

**FIGURE 1:**
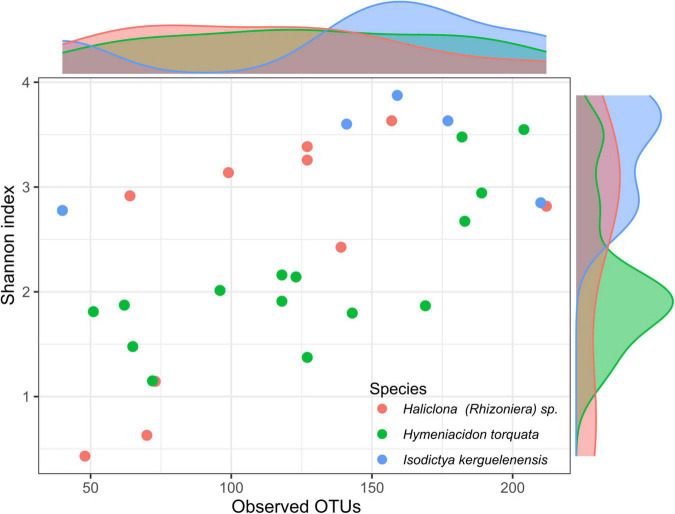
Richness and diversity patterns of Antarctic sponge-associated bacteria. Histograms at the top and right-hand side of the plot show the distribution of observed operational taxonomic units (OTUs) and Shannon index, respectively, for the individuals of the three sponge species.

The analysis of the sponge microbiomes yielded an average of 8,211 ± 7,508 sequences per sponge individual and a total of 359 different OTUs. Among observed OTUs per sponge individual, *I. kerguelenensis* had an average of 145 ± 64. *H. torquata* had an average of 126 ± 50, while *Haliclona (Rhizoniera)* sp. had an average of 111 ± 50 OTUs (Figure 1).

Mean Shannon index values were 2.1 ± 0.7 in *H. torquata*, 2.4 ± 1.2 in *Haliclona (Rhizoniera)* sp., and 3.43 ± 0.5 in *I. kerguelenensis*. ANOVA based on the Shannon index showed significant differences between sponge species (*F* = 3.48, *p* < 0.05). Differences between sponge species using the Tukey’s HSD test showed the only two species with significant differences for the Shannon index were *H. torquata* and *I. kerguelenensis* (confidence interval: 0.07–2.33, *p* = 0.04).

### Community Composition Patterns in Antarctic Sponge Microbiomes

**FIGURE 2:**
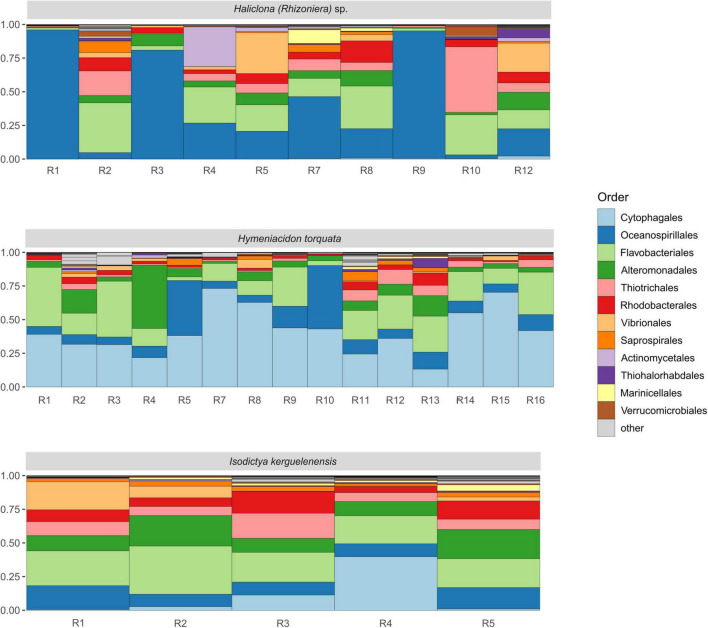
Bacterial composition of the microbiome of the Antarctic sponges *Haliclona (Rhizoniera)* sp., *Hymeniacidon torquata*, and *Isodictya kerguelenensis*, at order level. R followed by a number represents the individual from each sponge species. The color legend shows the 12 most abundant bacterial orders.

The taxonomic characterization of the Antarctic sponge bacterial symbionts revealed the presence of 11 different phyla (Supplementary Table 2), with Gammaproteobacteria, Flavobacteriia, and Cytophagia as dominant classes. Individuals R1, R3, and R9 of *Haliclona (Rhizoniera)* sp. showed a higher abundance of Oceanospirillales (more than 75%). At the same time, R10, R2, R4, R5, and R8 had a higher presence of Flavobacteriales and, in the case of R10, a higher dominance of Thiotrichales (Figure 2). R5 and R12 showed ∼25% of Vibrionales, while R4 was the only individual with a high abundance of Actinomycetales. Among the 15 individuals of *H. torquata*, the microbial community was overall dominated by Cytophagales with R5 and R10 also presenting a high abundance of Oceanospirillales, while R4 had a higher presence of Alteromonadales. Individuals of *I. kerguelenensis* showed a taxonomic composition characterized by Flavobacteriales and Alteromonadales in similar abundance across individuals. Cytophagales were higher in R4, while other main taxa (Rhodobacterales, Vibrionales, and Oceanospirillales) were overall even across individuals.

**FIGURE 3:**
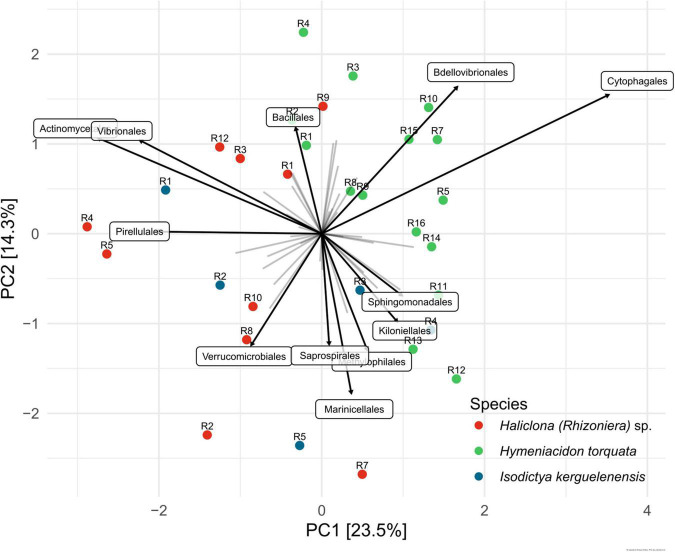
Ordination plot derived from unconstrained principal component analysis (PCA) of sponge-associated bacterial communities. R followed by a number on top of the colored dots represents the individual from each sponge species. The ordination includes the 12 bacterial orders with the highest significance to the microbiome variation between sponge individuals. The strength of the contribution is represented by the length of the arrow.

Unconstrained PCA showed that the bacterial community composition of *I. kerguelenensis* was similar with both *H. torquata* and *Haliclona (Rhizoniera)* sp., while individuals of *H. torquata* showed the highest similarity between them (Figure 3). Among the main bacterial orders shaping differences in the community, Cytophagales and Bdellovibrionales were the main taxa for *H. torquata*. Individuals of *H. torquata*, R1, R2, R3, and R4 were more similar to R1, R3, R9, and R12 of *Haliclona (Rhizoniera)* sp. due to the presence of Bacillales. Individuals of *I. kerguelenensis* cluster with *H. torquata* (R3 and R4) and with *Haliclona (Rhizoniera)* sp. (R1, R2, and R5), and their community is defined by a broader range of taxa. A detailed community composition at family and genus levels showed *Polaribacter* sp. and *Rhodobacteraceae* were similarly present among the three sponge species. At the same time, rare taxa (less than 1% of relative abundance) represent 28% of the microbiome of *I. kerguelenensis*, 19% of *Haliclona (Rhizoniera)* sp., and 12% of *H. torquata* (Supplementary Figure 1).

Differences in community composition of bacterial symbionts from these Antarctic sponges, determined by NPMANOVA (Supplementary Table 3), indicated *H. torquata* as the only species with a significant difference in OTU abundance profiles (*p*-value = 0.0001 for *H. torquata–Haliclona (Rhizoniera)* sp. and *p*-value = 0.0004 for *H. torquata–I. kerguelenensis*).

### Sponge Core Bacterial Community

**FIGURE 4:**
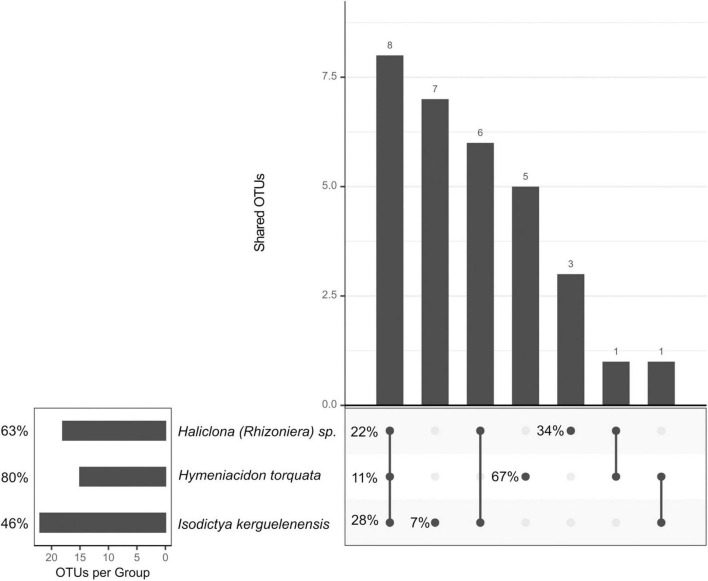
Upset plot showing the number of shared OTUs and representing the sponge core community. Percentages indicate the average contribution of those core OTUs to the microbial community relative abundance.

The “sponge core” microbiome was composed of 18 OTUs for *Haliclona (Rhizoniera)* sp., 15 for *H. torquata*, and 23 for *I. kerguelenensis* (Figure 4), representing 63, 80, and 46% of the total abundance, respectively. Among the sponge core OTUs, we identified eight OTUs shared across all sponge species (shared core OTUs). These OTUs corresponded to the orders Thiotrichales (OTU0008), Oceanospirillales (OTU0011 and OTU0027), Flavobacteriales (OTU0012), Rhodobacterales (OTU0016, OTU0020, and OTU0026), and Alteromonadales (OTU0028). However, the total abundance of these shared core OTUs was different between the three sponge species, with *I. kerguelenensis* showing the more abundant “shared core” community (28%) in comparison with *Haliclona (Rhizoniera)* sp. (22%) and *H. torquata* (11%).

*Isodictya kerguelenensis* had the higher number of species-specific core OTUs, with seven OTUs present across all individuals but representing only 7% of the symbionts’ relative abundance, while *H. torquata* had five OTUs representing 69% and *Haliclona (Rhizoniera)* sp. three OTUs corresponding to 34% of the sponge microbial community (details on Supplementary Table 4). The species-specific core bacterial community of *Haliclona (Rhizoniera)* sp. was composed of Oceanospirillales (OTU003), Actinomycetales (OTU0023), and Alteromonadales (OTU0077). In the case of *H*. *torquata*, it consisted of Cytophagales (OTU0001), Flavobacteriales (OTU0006), Oceanospirillales (OTU0007), Alteromonadales (OTU0010), and Rhodobacterales (OTU0065). For *I. kerguelenensis*, this community was composed by Alteromonadales (OTU0025 and OTU123), Flavobacteriales (OTU0031, OTU0056, OTU0113, and OTU0115), Thiotrichales (OTU0030), and Legionellales (OTU0159). Detailed OTU taxonomic assignment is available in Supplementary Table 5.

### Functional Potential of Bacterial Communities Associated With Antarctic Sponges

**FIGURE 5:**
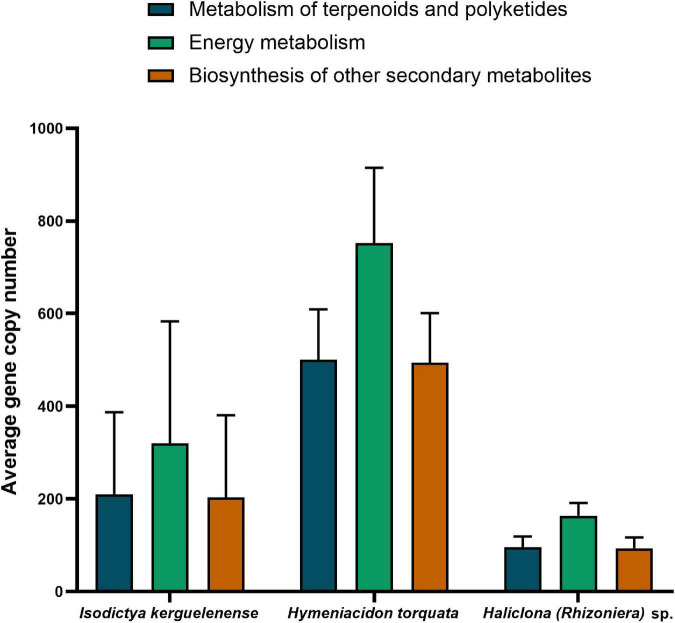
Average gene copy number predicted for each sponge species microbiome for pathways related to energy metabolism, metabolisms of terpenoids and polyketides, and biosynthesis of other secondary metabolites.

We screened the functional potential of the bacterial symbionts from the three Antarctic sponge species analyzed in this study to understand if the variability at the community composition within and between sponge species was maintained at the functional level. The microbiome of the three sponges showed a conserved functional potential with subtle differences in gene copy number of pathways related to energy metabolism, metabolisms of terpenoids and polyketides, and biosynthesis of other secondary metabolites (Figure 5). The NSTI, a value that expresses the availability of representative genomes for each microbiome, was similar between sponge species: 0.12 ± 0.028 for *Haliclona (Rhizoniera)* sp., 0.15 ± 0.002 for *H. torquata*, and 0.11 ± 0.03 for *I. kerguelenensis*.

*Hymeniacidon torquata* had the bacterial microbiome with the highest gene copy number of predicted functions related to metabolisms. In contrast, bacteria associated with *Haliclona (Rhizoniera)* sp. showed fewer gene copies involved in these functions. Specific predicted pathways involving energy metabolism (potential for nitrogen, sulfur, and methane metabolisms) also showed differences between species (Supplementary Table 6). The microbiome of *H. torquata* had the highest potential (referred here as higher gene copy number) for nitrogen metabolism (260 ± 56) and sulfur metabolism (180 ± 39).

## Discussion

In this study, we used 16S rRNA gene sequencing to study variations in the specificity of bacterial communities associated with three common sponge species from the WAP: *Haliclona (Rhizoniera)* sp., *H. torquata*, and *I. kerguelenensis*. We used a relatively high number of individuals per sponge species (10, 15, and 5, respectively) compared to previous studies on Antarctic sponges, allowing us to better resolve the specific traits representing each sponge microbiome.

Microbiome specificity in sponges, i.e., vertical microbiome transmission, has been extensively discussed, mainly regarding HMA and LMA sponges (Gloeckner et al., 2014; Erwin et al., 2015; De Mares et al., 2017; Moitinho-Silva et al., 2017). In most cases, LMA sponges are characterized by stable communities dominated by a few taxa, high temporal stability, and a higher degree of host specificity than HMA species (Erwin et al., 2015; Cárdenas et al., 2019). In this way, the community patterns detected in *Haliclona (Rhizoniera)* sp., *H. torquata*, and *I. kerguelenensis*, while agreeing with the microbiome inter-specificity of an LMA sponge (Rodríguez-Marconi et al., 2015; Cárdenas et al., 2018), still suggest intra-specific variations but with different degrees of specificity depending on the sponge species.

The differences observed in richness and diversity across sponge species suggest different contribution levels of less abundant OTUs over the microbiome of *I. kerguelenensis* and *Haliclona (Rhizoniera)* sp. Overall, the low OTU diversity observed in *H. torquata* is in accordance with previous studies on other Antarctic sponge species (Sacristán-Soriano et al., 2020). These observations also relate to unconstrained PCA results, which showed individuals of *H. torquata* clustering closer together and being defined by a narrowed community dominated by Bdellovibrionales and Cytophagales. Contrarily, *Haliclona (Rhizoniera)* sp. and *I. kerguelenensis* are structured by a greater repertoire of different taxa, some of them in low abundance. We also observed a dominance and co-occurrence of Bdellovibrionales, a predatory bacteria that help with the host energy requirements under low-nutrient conditions (Martínez et al., 2013; Welsh et al., 2016), and Cytophagales, a common gliding bacteria, on the microbiome of *H. torquata.* This trend has also been observed on coral microbiomes (Welsh et al., 2016), suggesting a common marine symbiotic relationship. These profiles suggest intra-specific differences in the microbiome across sponge species.

*Hymeniacidon torquata* displays the highest degree of specificity over their bacterial symbiotic community, with a core community representing 69% of the microbial abundance and corresponding only to five OTUs. This result suggest a lower degree of community acquisition from the surrounding seawater and, therefore, a reduced presence of transient bacteria. A similar analysis of 37 sponge species from different sites around the world oceans, but without including Antarctica, found that more than half of the detected OTUs were present only in a single sponge species (Schmitt et al., 2012). These authors suggest that sponge species share a small core community that is still different from the seawater and that the species-specific core community is probably vertically transmitted. In our case, this hypothesis seems plausible, with sponges sharing a small core community (eight shared core OTUs) that represents different abundances over sponge species microbiomes, suggesting a different selection over the taxa acquired from the surrounding seawater. In our study, the species-specific core community was also dominated by few OTUs per sponge species. These OTUs represented a larger proportion of the composition for *Haliclona (Rhizoniera)* sp. and *H. torquata*, while a very low proportion of *I. kerguelenensis*, indicating an overall specificity over this community that could be explained by vertical transmission but that is not equally conserved across sponge species.

Studies on LMA and HMA sponges showed that the contribution of the core community varies across sponge species, as well as the percentage of the core community that is acquired *via* seawater, supporting the existence of different strategies for microbiome acquisition across sponge species (Turon et al., 2018). Core OTUs described here showed eight OTUs shared across all sponge species. The community composition of these OTUs corresponds to Gammaproteobacteria, Alphaproteobacteria, and Flavobacteriia, all classes that have been described to be present in sponges and their surrounding seawater of this area (Rodríguez-Marconi et al., 2015). Therefore, this shared core community probably corresponds to bacterial members obtained from the seawater. The core OTUs had different contribution levels to the sponge microbiome, with the higher contribution observed on the microbiome of *I. kerguelenensis* and *Haliclona (Rhizoniera)* sp. (28 and 22%, respectively) in comparison with the 11% on *H. torquata*. These results agree with the lower diversity observed in *H. torquata* and the higher contribution that the species-specific core OTUs represent in this species.

In this work, we also provide an overview of the microbiome potential capacity of the Antarctic sponges over generic biochemical pathways. Although with several limitations, this approach has been widely used as an initial exploration of microbial functional potential in different environments (Cleary et al., 2017; Koo et al., 2017; Selvarajan et al., 2019), including temperate (de Voogd et al., 2015; Helber et al., 2019) and Antarctic sponge microbiomes (Steinert et al., 2019). Functional inferences based on conserved marker genes can be further used as a screening tool in metagenomic and metatranscriptomic studies to deepen the analyses of selected metabolic pathways.

Predicted functions of the bacterial microbiomes from the sponges analyzed in this study include pathways related to cellular processes, environmental information and genetic information processing, organismal systems, and metabolisms, with a highly conserved profile among individuals and sponge species. These categories represent broad functional categories previously identified in other Antarctic sponge microbiomes using a similar 16S rRNA gene-based functional approach (Steinert et al., 2019). In order to support our bioinformatics analysis, we corroborated that the functions predicted in this study were present in the only Antarctic sponge metagenomes available so far (Moreno-Pino et al., 2020).

All three sponge microbiomes analyzed in this study showed potential for nitrogen, sulfur, and methane metabolisms, with the microbiomes of *H. torquata* exhibiting the higher abundance of gene copies related to all these pathways and, in particular, to methane metabolism. Accordingly, *H. torquata* presented the highest abundance of the methane-oxidizing clade SUP05. This pathway was described in deep-sea sponges (Rubin-Blum et al., 2019), where, due to poor light availability, sponges present chemosynthetic bacterial symbionts with the capacity of oxidizing methane. A similar situation could occur in the microbiomes of Antarctic sponges as they are exposed to long periods of darkness during the Austral winter. Genes implicated in nitrogen fixation, nitrification, anaerobic respiration of ammonium, and denitrification have been frequently described in marine sponges from different environments (Wilkinson and Fay, 1979; Bayer et al., 2008; Hoffmann et al., 2009; Schläppy et al., 2010; Radax et al., 2012). These processes are among the main metabolisms in marine microorganisms with symbiotic lifestyles with invertebrate hosts (Fiore et al., 2010). In the case of the Antarctic sponges, *L. antarctica* and *Myxilla* sp., the archaea *Nitrosopumilus* sp. and the bacterial taxa Rhodospirillales were identified as involved in ammonia and nitrite oxidation and denitrification, although in different proportions in each sponge species (Moreno-Pino et al., 2020). These microorganisms were also identified in the microbiomes of the three sponges from this study but in an extremely low abundance, suggesting that rare microorganisms participate in main metabolism pathways. A similar case was demonstrated for the deep-sea sponge *Hexadella cf. dedritifera*, where symbiotic taxa present in low abundance might have great implications on functional traits (Reveillaud et al., 2014). Sulfur cycling has been described as an important pathway in sponges from cold environments (Jensen et al., 2017; Tian et al., 2017) and to be driven by Nitrosomonadales, also present in the sponge species here analyzed in low abundance.

Due to the great potential of sponges as a source for biomedical compounds (Ruocco et al., 2021; Varijakzhan et al., 2021), we also included pathways related to the biosynthesis of antibiotics (butirosin, neomycin, novobiocin, penicillin, cephalosporins, streptomycin, ansamycins, vancomycin group, and tetracyclines), besides genes associated with the biosynthesis of polyketide sugar units and carotenoids. These compounds are synthesized by routes of terpenoids, polyketides, and other secondary metabolites. We found genes related to the biosynthesis of important antimicrobial compounds in all the sponges analyzed, supporting previous studies demonstrating the enormous biotechnological potential behind the sponge microbiomes of extreme environments (Papaleo et al., 2012; Berne et al., 2016), as well as the importance to continue understanding and protecting this ecosystem.

## Conclusion

The Antarctic sponges *Haliclona (Rhizoniera)* sp., *H. torquata*, and *I. kerguelenensis* showed an overall conserved taxonomic profile across individuals of each sponge species with a distinct core community and differences in its contribution to the total microbiome. Taxonomic profiles show specific traits for each sponge species, particularly for *H. torquata*, which had the highest degree of specificity over their microbiome. Inference of the potential to express interesting pathways related to energy metabolisms, metabolisms terpenoids and polyketides, and biosynthesis of secondary metabolites indicates a functional convergence despite taxonomic differences in the bacterial communities. Overall, this study provides evidence to support that the relationship between the Antarctic sponge microbiome and their hosts is not equally conserved and suggests differences in the acquisition of the sponge microbiome among host species.

## Data Availability Statement

The datasets generated for this study can be found in the NCBI SRA as BioProject PRJNA611843 (https://www.ncbi.nlm.nih.gov/sra). All R coding and data used for the analyses is available in https://github.com/acristim/Antarctic-sponges.

## Ethics Statement

This study was conducted under permit 806/2015 granted by the Chilean Antarctic Institute (INACH).

## Author Contributions

CC and NT conceived the experiments. CC took the sponge samples. FM-V did the molecular procedures for amplicon sequencing and initial sequence processing. AC and GP-P conducted the bioinformatics analyses. AC and GP-P interpreted the data. AC, CC, and NT drafted the manuscript. All authors contributed to the article and approved the submitted version.

## Conflict of Interest

The authors declare that the research was conducted in the absence of any commercial or financial relationships that could be construed as a potential conflict of interest.

## Publisher’s Note

All claims expressed in this article are solely those of the authors and do not necessarily represent those of their affiliated organizations, or those of the publisher, the editors and the reviewers. Any product that may be evaluated in this article, or claim that may be made by its manufacturer, is not guaranteed or endorsed by the publisher.

## References

[B1] Adair K. L. Douglas A. E. (2017). Making a microbiome: the many determinants of host-associated microbial community composition. *Curr. Opin. Microbiol.* 35 23–29. 10.1016/j.mib.2016.11.002 27907842

[B2] Barnett D. Arts I. Penders J. (2021). microViz: an R package for microbiome data visualization and statistics. *J. Open Source Softw.* 6:3201. 10.21105/joss.03201

[B3] Bayer K. Schmitt S. Hentschel U. (2008). Physiology, phylogeny and in situ evidence for bacterial and archaeal nitrifiers in the marine sponge *Aplysina aerophoba*. *Environ. Microbiol.* 10 2942–2955. 10.1111/j.1462-2920.2008.01582.x 18363713

[B4] Bell J. J. (2008). The functional roles of marine sponges. *Estuar. Coast. Shelf Sci.* 79 341–353. 10.1016/j.ecss.2008.05.002

[B5] Berne S. Kalauz M. Lapat M. Savin L. Janussen D. Kersken D. (2016). Screening of the Antarctic marine sponges (Porifera) as a source of bioactive compounds. *Polar Biol.* 39 947–959. 10.1007/s00300-015-1835-4

[B6] Björk J. R. Díez-Vives C. Astudillo-García C. Archie E. A. Montoya J. M. (2019). Vertical transmission of sponge microbiota is inconsistent and unfaithful. *Nat. Ecol. Evol.* 3 1172–1183. 10.1038/s41559-019-0935-x 31285574 PMC6914380

[B7] Burke C. Steinberg P. Rusch D. Kjelleberg S. Thomas T. (2011). Bacterial community assembly based on functional genes rather than species. *Proc. Natl. Acad. Sci. U. S. A.* 108 14288–14293. 10.1073/pnas.1101591108 21825123 PMC3161577

[B8] Caporaso J. G. Lauber C. L. Walters W. A. Berg-Lyons D. Lozupone C. A. Turnbaugh P. J. (2011). Global patterns of 16S rRNA diversity at a depth of millions of sequences per sample. *Proc. Natl. Acad. Sci. U. S. A.* 108(SUPPL. 1) 4516–4522. 10.1073/pnas.1000080107 20534432 PMC3063599

[B9] Cárdenas C. A. Font A. Steinert G. Rondon R. González-Aravena M. (2019). Temporal stability of bacterial communities in Antarctic Sponges. *Front. Microbiol.* 10:2699. 10.3389/fmicb.2019.02699 31824467 PMC6883807

[B10] Cárdenas C. A. González-Aravena M. Font A. Hestetun J. T. Hajdu E. Trefault N. (2018). High similarity in the microbiota of cold- water sponges of the Genus Mycale from two different geographical areas. *PeerJ* 6:4935. 10.7717/peerj.4935 29892508 PMC5994334

[B11] Cleary D. F. R. Coelho F. J. R. C. Oliveira V. Gomes N. C. M. Polónia A. R. M. (2017). Sediment depth and habitat as predictors of the diversity and composition of sediment bacterial communities in an inter-tidal estuarine environment. *Mar. Ecol.* 38:e12411. 10.1111/maec.12411

[B12] De Goeij J. M. Van Oevelen D. Vermeij M. J. A. Osinga R. Middelburg J. J. De Goeij A. F. P. M. (2013). Surviving in a marine desert: the sponge loop retains resources within coral reefs. *Science* 342 108–110. 10.1126/science.1241981 24092742

[B13] De Mares M. C. Sipkema D. Huang S. Bunk B. Overmann J. van Elsas J. D. (2017). Host specificity for bacterial, archaeal and fungal communities determined for high- and low-microbial abundance sponge species in two genera. *Front. Microbiol.* 8:2560. 10.3389/fmicb.2017.02560 29326681 PMC5742488

[B14] de Voogd N. J. Cleary D. F. R. Polónia A. R. M. Gomes N. C. M. (2015). Bacterial community composition and predicted functional ecology of sponges, sediment and seawater from the thousand islands reef complex, West Java, Indonesia. *FEMS Microbiol. Ecol.* 91:fiv019. 10.1093/femsec/fiv019 25764467

[B15] Downey R. V. Griffiths H. J. Linse K. Janussen D. (2012). Diversity and distribution patterns in high Southern latitude sponges. *PLoS One* 7:e41672. 10.1371/journal.pone.0041672 22911840 PMC3404021

[B16] Erwin P. M. Coma R. López-Sendino P. Serrano E. Ribes M. (2015). Stable symbionts across the HMA-LMA dichotomy: low seasonal and interannual variation in sponge-associated bacteria from taxonomically diverse hosts. *FEMS Microbiol. Ecol.* 91:fiv115. 10.1093/femsec/fiv115 26405300

[B17] Fernandez J. C. C. Bravo-Gómez D. Cárdenas C. A. Hajdu E. (2020). Sponges from Doumer Island, Antarctic Peninsula, with description of new species of Clathria (Axosuberites) Topsent, 1893 and Hymeniacidon Bowerbank, 1858, and a re-description of H. Torquata Topsent, 1916. *Zootaxa* 4728 77–109. 10.11646/zootaxa.4728.1.4 32230585

[B18] Fiore C. L. Jarett J. K. Olson N. D. Lesser M. P. (2010). Nitrogen fixation and nitrogen transformations in marine symbioses. *Trends Microbiol.* 18 455–463. 10.1016/j.tim.2010.07.001 20674366

[B19] Fiore C. L. Labrie M. Jarett J. K. Lesser M. P. (2015). Transcriptional activity of the giant barrel sponge, Xestospongia muta Holobiont: molecular evidence for metabolic interchange. *Front. Microbiol.* 6:364. 10.3389/fmicb.2015.00364 25972851 PMC4412061

[B20] Gao Z. M. Wang Y. Tian R. M. Wong Y. H. Batang Z. B. Al-Suwailem A. M. (2014). Symbiotic adaptation drives genome streamlining of the cyanobacterial sponge symbiont “Candidatus Synechococcus spongiarum”. *MBio* 5:e00079-14. 10.1128/mBio.00079-14 24692632 PMC3977351

[B21] Gilbert J. A. Jansson J. K. Knight R. (2014). The Earth Microbiome project: successes and aspirations. *BMC Biol.* 12:69. 10.1186/s12915-014-0069-1 25184604 PMC4141107

[B22] Gloeckner V. Wehrl M. Moitinho-Silva L. Gernert C. Hentschel U. Schupp P. (2014). The HMA-LMA dichotomy revisited: an electron microscopical survey of 56 sponge species. *Biol. Bull.* 227 78–88. 10.1086/BBLv227n1p78 25216505

[B23] Hardoim C. C. P. Costa R. (2014). Temporal dynamics of prokaryotic communities in the marine sponge Sarcotragus spinosulus. *Mol. Ecol.* 23 3097–3112. 10.1111/mec.12789 24814756

[B24] Helber S. B. Steinert G. Wu Y. C. Rohde S. Hentschel U. Muhando C. A. (2019). Sponges from Zanzibar host diverse prokaryotic communities with potential for natural product synthesis. *FEMS Microbiol. Ecol.* 95:fiz026. 10.1093/femsec/fiz026 30830220

[B25] Hentschel U. Horn M. Friedrich A. B. Wagner M. Moore B. S. (2002). Molecular evidence for a uniform microbial community in sponges. *Appl. Environ. Microbiol.* 68 4431–4440. 10.1128/AEM.68.9.4431 12200297 PMC124103

[B26] Hoffmann F. Radax R. Woebken D. Holtappels M. Lavik G. Rapp H. T. (2009). Complex nitrogen cycling in the sponge *Geodia barretti*. *Environ. Microbiol.* 11 2228–2243. 10.1111/j.1462-2920.2009.01944.x 19453700

[B27] Jensen S. Fortunato S. A. V. Hoffmann F. Hoem S. Rapp H. T. Øvreås L. (2017). The relative abundance and transcriptional activity of marine sponge-associated microorganisms emphasizing groups involved in sulfur cycle. *Microb. Ecol.* 73 668–676. 10.1007/s00248-016-0836-3 27664049

[B28] Koo H. Mojib N. Hakim J. A. Hawes I. Tanabe Y. Andersen D. T. (2017). Microbial communities and their predicted metabolic functions in growth laminae of a unique large conical mat from Lake Untersee, East Antarctica. *Front. Microbiol.* 8:1347. 10.3389/fmicb.2017.01347 28824553 PMC5543034

[B29] Langille M. G. I. Zaneveld J. Caporaso J. G. McDonald D. Knights D. Reyes J. A. (2013). Predictive functional profiling of microbial communities using 16S rRNA marker gene sequences. *Nat. Biotechnol.* 31 814–821. 10.1038/nbt.2676 23975157 PMC3819121

[B30] Martínez V. Jurkevitch E. García J. L. Prieto M. A. (2013). Reward for *Bdellovibrio* bacteriovorus for preying on a polyhydroxyalkanoate producer. *Environ. Microbiol.* 15 1204–1215. 10.1111/1462-2920.12047 23227863

[B31] McMurdie P. J. Holmes S. (2013). Phyloseq: an R package for reproducible interactive analysis and graphics of microbiome census data. *PLoS One* 8:e61217. 10.1371/journal.pone.0061217 23630581 PMC3632530

[B32] Moitinho-Silva L. Steinert G. Nielsen S. Hardoim C. C. P. Wu Y. C. McCormack G. P. (2017). Predicting the HMA-LMA status in marine sponges by machine learning. *Front. Microbiol.* 8:752. 10.3389/fmicb.2017.00752 28533766 PMC5421222

[B33] Moreno-Pino M. Cristi A. Gillooly J. F. Trefault N. (2020). Characterizing the microbiomes of Antarctic sponges: a functional metagenomic approach. *Sci. Rep.* 10:645. 10.1038/s41598-020-57464-2 31959785 PMC6971038

[B34] Moreno-Pino M. Ugalde J. A. Valdés J. H. Rodríguez-Marconi S. Parada-Pozo G. Trefault N. (2021). Bacteria isolated from the antarctic sponge iophon sp. reveals mechanisms of symbiosis in sporosarcina, cellulophaga, and nesterenkonia. *Front. Microbiol.* 12:660779. 10.3389/fmicb.2021.660779 34177840 PMC8222686

[B35] Papaleo M. C. Fondi M. Maida I. Perrin E. Lo Giudice A. Michaud L. (2012). Sponge-associated microbial Antarctic communities exhibiting antimicrobial activity against Burkholderia cepacia complex bacteria. *Biotechnol. Adv.* 30 272–293. 10.1016/j.biotechadv.2011.06.011 21742025

[B36] Pita L. Rix L. Slaby B. M. Franke A. Hentschel U. (2018). The sponge holobiont in a changing ocean: from microbes to ecosystems. *Microbiome* 6:46. 10.1186/s40168-018-0428-1 29523192 PMC5845141

[B37] Radax R. Hoffmann F. Rapp H. T. Leininger S. Schleper C. (2012). Ammonia-oxidizing archaea as main drivers of nitrification in cold-water sponges. *Environ. Microbiol.* 14 909–923. 10.1111/j.1462-2920.2011.02661.x 22176665

[B38] Reveillaud J. Maignien L. Eren M. A. Huber J. A. Apprill A. Sogin M. L. (2014). Host-specificity among abundant and rare taxa in the sponge microbiome. *ISME J.* 8 1198–1209. 10.1038/ismej.2013.227 24401862 PMC4030224

[B39] Ribes M. Jiménez E. Yahel G. López-Sendino P. Diez B. Massana R. (2012). Functional convergence of microbes associated with temperate marine sponges. *Environ. Microbiol.* 14 1224–1239. 10.1111/j.1462-2920.2012.02701.x 22335606

[B40] Rodríguez-Marconi S. De La Iglesia R. Díez B. Fonseca C. A. Hajdu E. Trefault N. (2015). Characterization of bacterial, archaeal and eukaryote symbionts from antarctic sponges reveals a high diversity at a three-domain level and a particular signature for this ecosystem. *PLoS One* 10:e0138837. 10.1371/journal.pone.0138837 26421612 PMC4589366

[B41] Rubin-Blum M. Antony C. P. Sayavedra L. Martínez-Pérez C. Birgel D. Peckmann J. (2019). Fueled by methane: deep-sea sponges from asphalt seeps gain their nutrition from methane-oxidizing symbionts. *ISME J.* 13 1209–1225. 10.1038/s41396-019-0346-7 30647460 PMC6474228

[B42] Ruocco N. Esposito R. Bertolino M. Zazo G. Sonnessa M. Andreani F. (2021). A metataxonomic approach reveals diversified bacterial communities in Antarctic Sponges. *Mar. Drugs* 19:173. 10.3390/md19030173 33810171 PMC8004616

[B43] Sacristán-Soriano O. Pérez Criado N. Avila C. (2020). Host species determines symbiotic community composition in Antarctic Sponges (Porifera: Demospongiae). *Front. Mar. Sci.* 7:474. 10.3389/fmars.2020.00474

[B44] Savoca S. Lo Giudice A. Papale M. Mangano S. Caruso C. Spanò N. (2019). Antarctic sponges from the Terra Nova Bay (Ross Sea) host a diversified bacterial community. *Sci. Rep.* 9:16135. 10.1038/s41598-019-52491-0 31695084 PMC6834628

[B45] Schläppy M. L. Schöttner S. I. Lavik G. Kuypers M. M. M. de Beer D. Hoffmann F. (2010). Evidence of nitrification and denitrification in high and low microbial abundance sponges. *Mar. Biol.* 157 593–602. 10.1007/s00227-009-1344-5 24391241 PMC3873014

[B46] Schloss P. D. Westcott S. L. Ryabin T. Hall J. R. Hartmann M. Hollister E. B. (2009). Introducing mothur: open-source, platform-independent, community-supported software for describing and comparing microbial communities. *Appl. Environ. Microbiol.* 75 7537–7541. 10.1128/AEM.01541-09 19801464 PMC2786419

[B47] Schmitt S. Angermeier H. Schiller R. Lindquist N. Hentschel U. (2008). Molecular microbial diversity survey of sponge reproductive stages and mechanistic insights into vertical transmission of microbial symbionts. *Appl. Environ. Microbiol.* 74 7694–7708. 10.1128/AEM.00878-08 18820053 PMC2607154

[B48] Schmitt S. Tsai P. Bell J. Fromont J. Ilan M. Lindquist N. (2012). Assessing the complex sponge microbiota: core, variable and species-specific bacterial communities in marine sponges. *ISME J.* 6 564–576. 10.1038/ismej.2011.116 21993395 PMC3280146

[B49] Selvarajan R. Sibanda T. Venkatachalam S. Ogola H. J. O. Christopher Obieze C. Msagati T. A. (2019). Distribution, interaction and functional profiles of epiphytic bacterial communities from the rocky intertidal seaweeds, South Africa. *Sci. Rep.* 9:19835. 10.1038/s41598-019-56269-2 31882618 PMC6934600

[B50] Steinert G. Wemheuer B. Janussen D. Erpenbeck D. Daniel R. Simon M. (2019). Prokaryotic diversity and community patterns in antarctic continental shelf sponges. *Front. Mar. Sci.* 6:297. 10.3389/fmars.2019.00297

[B51] Taylor M. W. Schupp P. J. Dahllöf I. Kjelleberg S. Steinberg P. D. (2004). Host specificity in marine sponge-associated bacteria, and potential implications for marine microbial diversity. *Environ. Microbiol.* 6 121–130. 10.1046/j.1462-2920.2003.00545.x 14756877

[B52] Thiel V. Leininger S. Schmaljohann R. Brümmer F. Imhoff J. F. (2007). Sponge-specific bacterial associations of the Mediterranean sponge Chondrilla nucula (Demospongiae, Tetractinomorpha). *Microb. Ecol.* 54 101–111. 10.1007/s00248-006-9177-y 17364249

[B53] Thomas T. Moitinho-Silva L. Lurgi M. Björk J. R. Easson C. Astudillo-García C. (2016). Diversity, structure and convergent evolution of the global sponge microbiome. *Nat. Commun.* 7:11870. 10.1038/ncomms11870 27306690 PMC4912640

[B54] Tian R.-M. Zhang W. Cai L. Wong Y.-H. Ding W. Qian P.-Y. (2017). Genome reduction and microbe-host interactions drive adaptation of a sulfur-oxidizing bacterium associated with a cold seep sponge. *mSystems* 2:e00184-16. 10.1128/msystems.00184-16 28345060 PMC5361782

[B55] Turon M. Cáliz J. Garate L. Casamayor E. O. Uriz M. J. (2018). Showcasing the role of seawater in bacteria recruitment and microbiome stability in sponges. *Sci. Rep.* 8:15201. 10.1038/s41598-018-33545-1 30315194 PMC6185911

[B56] Varijakzhan D. Loh J. Y. Yap W. S. Yusoff K. Seboussi R. Lim S. H. E. (2021). Bioactive compounds from marine sponges: fundamentals and applications. *Mar. Drugs* 19:246. 10.3390/md19050246 33925365 PMC8146879

[B57] Webster N. S. Taylor M. W. (2012). Marine sponges and their microbial symbionts: love and other relationships. *Environ. Microbiol.* 14 335–346. 10.1111/j.1462-2920.2011.02460.x 21443739

[B58] Webster N. S. Thomas T. (2016). The sponge hologenome. *mBio* 7:e00135-16. 10.1128/mBio.00135-16 27103626 PMC4850255

[B59] Welsh R. M. Zaneveld J. R. Rosales S. M. Payet J. P. Burkepile D. E. Thurber R. V. (2016). Bacterial predation in a marine host-associated microbiome. *ISME J.* 10 1540–1544. 10.1038/ismej.2015.219 26613338 PMC5029193

[B60] Wilkinson C. R. Fay P. (1979). Nitrogen fixation in coral reef sponges with symbiotic cyanobacteria. *Nature* 279 527–529. 10.1038/279527a0

[B61] Zhang F. Blasiak L. C. Karolin J. O. Powell R. J. Geddes C. D. Hill R. T. (2015). Phosphorus sequestration in the form of polyphosphate by microbial symbionts in marine sponges. *Proc. Natl. Acad. Sci. U. S. A.* 112 4381–4386. 10.1073/pnas.1423768112 25713351 PMC4394258

[B62] Zhang S. Song W. Wemheuer B. Reveillaud J. Webster N. Thomas T. (2019). Comparative genomics reveals ecological and evolutionary insights into sponge-associated Thaumarchaeota. *mSystems* 4:e00288-19. 10.1128/msystems.00288-19 31409660 PMC6697440

