# Supplementary material for: Variability in Host Specificity and Functional Potential of Antarctic Sponge-Associated Bacterial Communities
Source: Front Microbiol. 2022 Jan 13;12:771589. doi: 10.3389/fmicb.2021.771589 (PMC8792898; doi:10.3389/fmicb.2021.771589)
Supplement: Supplementary file 2 [file Image_1.pdf]

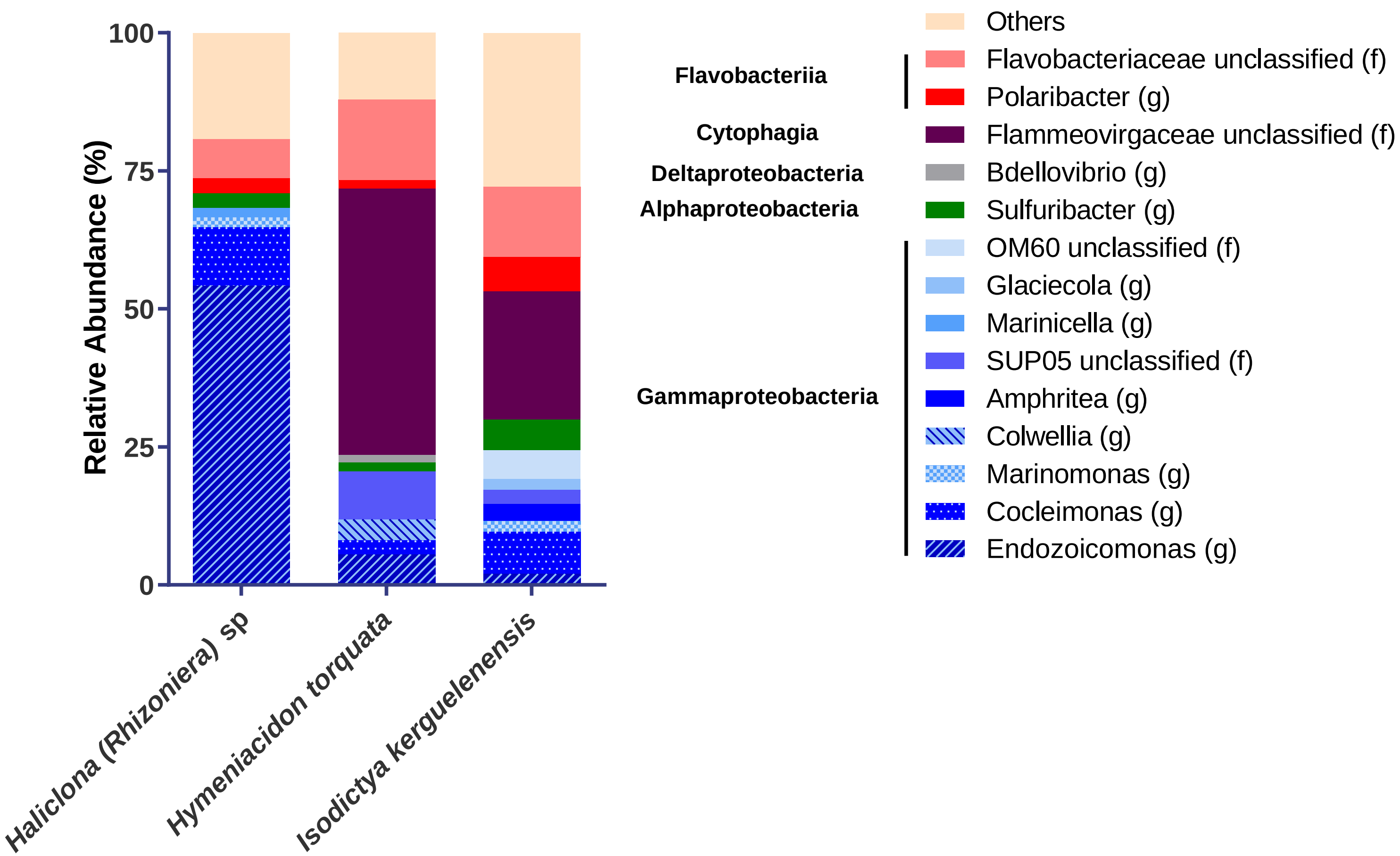

Supplementary Figure S1. Average taxonomic composition and Genus (g) and Family (f) level according to the deepest taxonomic assignation available for the microbiome of *Haliclona (Rhizoniera)* sp., *Hymeniacidon torquata* and *Isodictya kerguelenensis* with the corresponding taxonomic assignment at Class level.
